# Toxicity and medical countermeasure studies on the organophosphorus nerve agents VM and VX

**DOI:** 10.1098/rspa.2014.0891

**Published:** 2015-04-08

**Authors:** Helen Rice, Christopher H. Dalton, Matthew E. Price, Stuart J. Graham, A. Christopher Green, John Jenner, Helen J. Groombridge, Christopher M. Timperley

**Affiliations:** 1 Biomedical Sciences Department, Dstl Porton Down, Salisbury SP4 0JQ, UK; 2 Detection Department, Dstl Porton Down, Salisbury SP4 0JQ, UK

**Keywords:** chemical weapons, nerve agent, VX, VM, percutaneous penetration

## Abstract

To support the effort to eliminate the Syrian Arab Republic chemical weapons stockpile safely, there was a requirement to provide scientific advice based on experimentally derived information on both toxicity and medical countermeasures (MedCM) in the event of exposure to VM, VX or VM–VX mixtures. Complementary *in vitro* and *in vivo* studies were undertaken to inform that advice. The penetration rate of neat VM was not significantly different from that of neat VX, through either guinea pig or pig skin *in vitro*. The presence of VX did not affect the penetration rate of VM in mixtures of various proportions. A lethal dose of VM was approximately twice that of VX in guinea pigs poisoned via the percutaneous route. There was no interaction in mixed agent solutions which altered the *in vivo* toxicity of the agents. Percutaneous poisoning by VM responded to treatment with standard MedCM, although complete protection was not achieved.

## 1. Introduction

In October 2013, the Organisation for the Prohibition of Chemical Weapons (OPCW) was awarded the Nobel Peace Prize for its ‘extensive efforts to eliminate chemical weapons’ [1]. This, combined with efforts to destroy the Syrian Arab Republic's chemical weapons, brought the work of the OPCW and the Chemical Weapons Convention (CWC) [2] increased global attention. The highest priority for the OPCW over the last year has been addressing the Syrian Arab Republic's chemical weapon programme [3,4].

The third 5 yearly review conference for the CWC was held in The Hague in April 2013 [5]. There was a general expectation that it would mark the beginning of a new phase for the Convention—an evolution from the initial period of focus on destruction of existing chemical weapons to a new period of focus on preventing the re-emergence of chemical weapons. The bulk of the world's declared chemical weapon stockpiles had by then been destroyed, even though challenges remained. But the expectations turned out to be very premature. Concern was raised over the increasingly frequent and persuasive evidence that chemical weapons had been used in the Syrian Arab Republic by the Assad regime. The UN Secretary General had launched a formal investigation, led by Dr Sellström and staffed largely by the Technical Secretariat of the OPCW, and more evidence was gathered over the following months. The decisive event was the use of sarin in Damascus on 21 August 2013, resulting in the deaths of some 1500 civilians [6]. The UN investigation was able to conclude, based on a range of evidence, including analysis of samples, that sarin had been used [7,8]. Within weeks of the event in Damascus, an agreement between US Secretary Kerry and Russian Foreign Minister Lavrov prompted the Syrian Arab Republic to join the CWC [9]. The world was suddenly faced with a new chemical weapon destruction challenge.

But in one respect, this was less than in other cases: most CW possessor states had declared stocks which included filled munitions, often with explosive components, largely comprising actual CW agent. Destroying these stocks had required the building of specialized new destruction facilities: a complex, time-consuming and expensive process. Implementing such a programme in the Syrian Arab Republic would have been almost impossible. Fortunately, the Syrian Arab Republic stockpile was unique in comprising almost entirely precursor chemicals. This feature made it feasible to remove the chemicals from the Syrian Arab Republic for destruction elsewhere.

**Figure 1.**
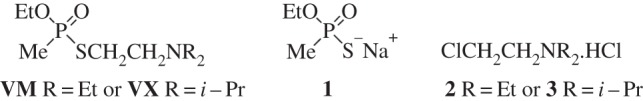
Structures of VM, VX and precursor chemicals: mixing **1** with **2**, or **3**, in aqueous solution produces the corresponding nerve agents, which pose a toxic hazard. The separate precursors have relatively low acute toxicity compared with the nerve agents.

The Syrian Arab Republic government declared approximately 1000 tonnes of chemical weapons, mostly precursors, and approximately 290 tonnes of raw materials [10]. The blister agent sulfur mustard was the only complete or unitary chemical warfare agent declared. The nerve agents sarin, VM and VX (figure 1) were stockpiled in binary form, meaning two precursors are stored and mixed just before use. The destruction of these chemicals was a multinational responsibility. The most hazardous—sulfur mustard (19.8 tonnes) and methylphosphonic difluoride (a sarin precursor, 581.5 tonnes)—were destroyed aboard the US naval ship Cape Ray [11]. Around 130 tonnes of an aqueous solution of sodium *O*-ethyl methylphosphonothioate **1**, the first precursor to VM and VX, were incinerated in Finland. Approximately 150 tonnes of the second precursors needed to make VM or VX have been incinerated by the British company Veolia [12]. The VM precursor, *N*,*N*-diethylaminoethyl-2-chloride hydrochloride **2**, was in aqueous solution and the VX precursor, *N*,*N*-diisopropylaminoethyl-2-chloride hydrochloride **3**, was stored separately in the solid state and aqueous solution.

VX and VM are highly potent nerve agents that act primarily by inhibiting the nerve agent acetylcholinesterase; V agents are typically much less volatile than G agents, like sarin, therefore absorption through the skin represents a particularly hazardous route of exposure for V agents. There are no reported studies on VM toxicology or responsiveness to medical countermeasures (MedCM), in contrast to VX, which has been shown to be responsive to treatment following poisoning by inhalation or skin contact [13,14]. It is essential to be able to provide scientific advice based on experimentally derived information on both toxicity and MedCM in the event of exposure to VM, VX or VM–VX mixtures, for destruction purposes (in case personnel encounter a situation where precursor **1** has mixed with precursor **2** and/or **3**). In response to the international effort to eliminate the Syrian Arab Republic stockpile safely, we conducted complementary *in vitro* and *in vivo* studies. Here, we present the first comparison of the percutaneous toxicity of VM and VX, and their mixtures, in a guinea pig model of nerve agent poisoning [15]. We also report the first study of the responsiveness of animals poisoned by VM to traditional nerve agent MedCM. We measured the percutaneous penetration rate of VM alone and mixed in various proportions with VX, in both guinea pig and pig skin. The collection of data from both species *in vitro* enables greater confidence in the extrapolation of results from the *in vivo* experiments to humans. These data, in combination with work conducted by other international partners on the acute toxicity of VM and its responsiveness to MedCM, will inform the hazard to humans from these nerve agents and their mixtures, and suitable medical approaches in case of accidental poisoning by them.

## 2. Material and methods

### (a) Synthesis of ^14^C VM and ^14^C VX

**Figure 2.**
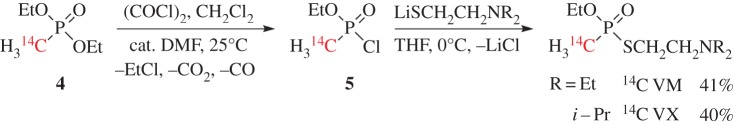
Synthesis of ^14^C VM and ^14^C VX in two steps. The unlabelled nerve agents (not shown) were prepared similarly from unlabelled diethyl methylphosphonate. (Online version in colour.)

The synthesis of these two nerve agents (figure 2) was accomplished using standard organophosphorus chemistry [16] after successful practice runs with unlabelled starting materials, by chlorination of diethyl [^14^C]-methylphosphonate **4** to give ethyl [^14^C]-methylphosphonochloridate **5**, followed by its reaction with lithium *N*,*N*-diethyl- or *N*,*N*-diisopropylaminoethane-2-thiolate. A solution of (EtO)_2_P(O)^14^CH_3_ (0.25 ml, 1.72 mmol; Quotient Bioresearch, Cardiff, South Glamorgan, UK; specific activity 58 mCi mmol^−1^) in dichloromethane (3 ml) and *N*,*N*-dimethylformamide (DMF, 0.005 ml) was treated with oxalyl chloride (0.18 ml, 2.10 mmol). The mixture effervesced and was stirred at room temperature for 12 h. The solvent was removed under reduced pressure to yield EtO(^14^CH_3_)P(O)Cl as a pale yellow liquid (0.25 g, 100%; approx. 98% pure by ^31^P NMR spectroscopy, its analytical constants agreed with literature data [17,18]). The entire portion of the chloridate was dissolved in anhydrous tetrahydrofuran (THF, 3 ml) and stored under nitrogen ready for the next step.

*n*-Butyllithium (0.68 ml, 1.70 mmol, 2.5 M in hexane) was added dropwise to a stirred solution of *N*,*N*-diethylaminoethane-2-thiol (0.23 g, 1.70 mmol) or *N*,*N*-diisopropylaminoethane-2-thiol (0.27 g, 1.70 mmol) in anhydrous THF (3 ml) at 0°C under nitrogen. The resulting solution was transferred by cannula to the chloridate solution (0.25 g, 1.72 mmol, in 3 ml of THF) and left to stir at 0°C for 1 h. The mixture was quenched with 10% hydrochloric acid (5 ml). The organic phase was separated and washed with water (3×2 ml). The aqueous washings were combined with the original aqueous phase and extracted with diethyl ether (3×4 ml). The aqueous phase containing the V-agent hydrochloride salt was basified with 2 M sodium hydroxide (2 ml) and re-extracted with chloroform (3×4 ml). The chloroform extracts were combined and dried (MgSO_4_). The drying agent was filtered off and the solvent evaporated under reduced pressure to give a liquid. This was purified by bulb-to-bulb distillation under vacuum to give ^14^C VM (0.17 g, 41%; oven temperature 120°C/0.001 mmHg, lit. bp 68°C/0.007 mmHg*) or ^14^C VX (0.18 g, 40%; oven temperature bp 180°C/0.001 mmHg, lit. bp 97°C/0.005 mmHg*) as colourless liquids. (Boiling points marked by an asterisk are those obtained by fractionation of unlabelled samples of the V-agents on a larger scale: however, boiling points of compounds of this type are an unreliable criterion of purity, as they can vary as much as 10°C according to the distillation rate).

The purities of the compounds were determined in CDCl_3_ solution by NMR spectroscopy at 9.4 Tesla using a Bruker Avance III spectrometer equipped with a 5 mm BBFO+ probehead.

#### (i) ^14^C VM

This was 98% pure by ^1^H, and 99% pure by ^31^P, NMR spectroscopy. ^1^H NMR (400 MHz): *δ* 4.26–4.06 (2H, m, OCH_2_), 3.03–2.86 (2H, m, SCH_2_), 2.83–2.71 (2H, m, CH_2_), 2.59 (4H, q, ^3^*J*_HH_=6.9 Hz, N(CH_2_CH_3_)_2_), 1.82 (3H, d, ^2^*J*_PH_=15.6 Hz, PCH_3_), 1.37 (3H, t, ^3^*J*_HH_=7.0 Hz, CH_3_), 1.07 (6H, t, ^3^*J*_HH_=7.0 Hz, N(CH_2_CH_3_)_2_) ppm. ^31^P{^1^H} NMR (161 MHz): *δ* 54.0 ppm.

#### (ii) ^14^C VX

This compound was 99% pure by both ^1^H NMR and ^31^P NMR spectroscopy. ^1^H NMR (400 MHz): *δ* 4.26–4.06 (2H, m, OCH_2_), 3.08–2.98 (2H, m, SCH_2_), 2.88–2.77 (2H, m, CH_2_), 2.73–2.68 (2H, m, N(CH(CH_3_)_2_)_2_), 1.81 (3H, d, ^2^*J*_PH_=15.3 Hz, PCH_3_), 1.37 (3H, t, ^3^*J*_HH_=7.0 Hz, CH_3_), 1.03 (12H, d, ^3^*J*_HH_=6.8 Hz, N(CH(CH_3_)_2_)_2_) ppm. ^31^P{^1^H} NMR (161 MHz): *δ* 54.3 ppm.

Labelled and unlabelled samples of the V-agents were confirmed analytically pure by mass spectrometry techniques that are reported elsewhere [19–24]. These are accredited under ISO/IEC 17 025:2005 and used routinely within the UK's OPCW Designated Laboratory at Dstl Porton Down. Samples of unlabelled VM and VX [25] of 99% purity, as indicated by NMR spectroscopy, had densities of 1.0311 and 1.0083 g ml^−1^ at 25°C and ether–water partition coefficients of 1.7 and 20.5 at 25°C.

### (b) *In vitro* skin penetration

Liquid scintillation counting (LSC) materials (soluene-350^TM^, ultima gold cocktail and opaque plastic vials) were purchased from Perkin-Elmer (Chandler's Ford, Hampshire, UK). All other chemicals were analytical grade and were purchased from VWR (Lutterworth, Leicestershire, UK).

Male Dunkin–Hartley guinea pigs (Harlan UK Ltd, Bicester, UK) were humanely killed, the dorsal (thoracic) skin close clipped, removed and stored flat at −20°C. Eight Large White pigs were humanely killed, the whole abdominal skin flank close clipped and excised. Skin sections were stored flat and frozen for a period not exceeding six months. After defrosting, skin samples were dermatomed to a nominal thickness of 500 μm using a Humeca dermatome (Model D42, Eurosurgical Ltd, Guildford, UK), prior to mounting in diffusion cells.

Percutaneous absorption experiments were performed with Franz-type glass diffusion cells as previously described [26]. Each lower (receptor) chamber was filled with 5±0.5 ml of 50% aqueous ethanol (v/v) (receptor fluid). Donor chambers had an available surface area of 2.54 cm^2^. The skin surface within each diffusion cell was maintained at a temperature of 32°C.

As ^14^C VX and ^14^C VM were used, each study was run twice, first to allow quantification of ^14^C VX penetration (used in combination with unlabelled VM) and second to allow quantification of ^14^C VM penetration (used in combination with unlabelled VX). The results from the two studies were combined to allow quantification of the combined ^14^C VX and ^14^C VM penetration rate. In all cases, agent combinations were applied as discrete 10 μl droplets onto the surface of each skin with the composition of each 10 μl droplet varying according to study type. Each mixture proportion was repeated using dilute agent (in isopropyl alcohol, IPA) for comparison with the *in vivo* guinea pig studies where agent was applied in a carrier solvent due to the extremely small volumes required. In each case the total concentration was 22 mg ml^−1^, with the proportions of VM and VX varying depending on the mixture being studied.

For all dosing conditions, the donor chambers were left unoccluded for the duration of the study. Each study used skin samples from eight animals to give *n*=8 biological replicates for each mixture proportion. Following application of the agent, samples (50 μl) of receptor chamber fluid were removed at regular intervals up to 24 h post exposure. Each sample was replaced with an equivalent volume of fresh receptor fluid. At the end of each experiment, each skin surface was gently swabbed with cotton wool which was then immersed in 20 ml of 50% aqueous ethanol. Each skin sample was dissolved in 10 ml of Soluene-350 and radioactivity measured using a Perkin-Elmer Tri-Carb liquid scintillation counter. The amount of radioactivity was converted to amount of VX and VM by comparison to standard samples prepared and measured simultaneously.

Maximum penetration rates ($J_{\max}$) were calculated by measuring the gradient of the line obtained by plotting the amount of agent penetrating against time (under finite dose conditions). Maximum penetration rates were expressed as the percentage of applied dose penetration rate in order to perform statistical comparisons between each mixture proportion.

### (c) *In vivo* experiments

All experiments were conducted according to the terms and conditions of a project licence (PPL 30/2864) issued by the UK Home Office under the Animals (Scientific Procedures) Act 1986. VX and VM were synthesized by Detection Department, Dstl, and supplied at >98% purity at a range of concentrations and mixture proportions in IPA.

Male HsdDhl:DH Dunkin–Hartley guinea pigs (Harlan, UK) were kept in standardized conditions throughout the study, according to UK Home Office guidelines (room temperature 21°C, humidity 50%). The lights were on from 06.00 to 18.00 h. Body weight and temperature were recorded daily throughout the experiment.

On each experimental day, VM, VX or a mixed solution were diluted to the appropriate concentration in IPA and applied to a clipped area of the dorsal skin at a dose volume of 0.033 ml kg^−1^. Nerve agent dosing was carried out in a fume cupboard in which the animals remained for 6 h under continuous observation. Guinea pigs were then returned to a laboratory and observed periodically thereafter. Clinical signs of nerve agent poisoning, e.g. tremor, incapacitation, lachrymation and fasciculations, were scored during the observation periods. Incapacitation was classified as normal, mild, moderate or substantial [27]. Survival was determined at 24 h, at which point the experiment ended and surviving animals were euthanized with an overdose of pentobarbital.

#### (i) *In vivo* toxicity

The 24 h percutaneous toxicity of VM alone, VX alone and three mixed solutions of VM and VX was determined using an adaptive design [28,29]. Apart from 100% VM, where two determinations of LD_50_ were carried out, only a single LD_50_ determination was made for the mixtures; therefore, no indication was obtained of the variance in the LD_50_.

#### (ii) *In vivo* MedCM

Guinea pigs were challenged via the percutaneous route, as above, with an approximately equitoxic (2×LD_50_) dose of either VM or VX (2.388 mg kg^−1^ or 1.226 mg kg^−1^, respectively, as determined in the toxicity experiments). Therapy of atropine sulfate (Sigma UK), Avizafone dihydrochloride (Roche UK) and either P2S (pralidoxime methanesulphonate) (Dstl oxime stock) or HI-6 dimethanesulphonate (Edinburgh Pharmaceutical Processes, UK) was administered (i.m.) on the appearance of signs of cholinergic poisoning and subsequently on worsening signs of poisoning up to a maximum of three doses. The total therapy doses were P2S 30 mg kg^−1^ or HI-6 30 mg kg^−1^; atropine 17.4 mg kg^−1^; Avizafone 3.14 mg kg^−1^. These doses were selected to align with doses we have previously used in guinea pigs [15], and the oxime doses were not equimolar to each other or to human autoinjector doses.

### (d) Statistical analysis

Skin penetration rates (expressed as percentage of applied dose penetration rates) were compared using a non-parametric ANOVA (Kruskal–Wallis test) with Dunn's multiple comparisons post test in Graphpad Prism 5 (GraphPad Software Inc.).

A probit regression model was used to fit a dose–response curve to the survival data using the generalized linear model in the R statistical software package. The LD_50_ was calculated from the probit regression by rearranging the model formula in terms of dose. Approximate standard errors for fit to this dose estimate were calculated using the transformation method and confidence intervals using the delta method. At the conclusion of the study all LD_50_ values were reanalysed using a bias reduced logistic regression fitting method [30,31]. The predicted toxicity of mixtures of VM and VX was calculated from a simple proportional combination [32] using, for VM, the estimate of the LD_50_ derived from analysis of the combined data set of both VM-alone determinations (1.2899 mg kg^−1^), and a value for VX derived from a recent determination in our laboratory. The 95% CI envelope was constructed from the highest and lowest 95% CI on the two independent determinations of VM toxicity.

In the MedCM studies, the primary end points were 6 h survival and 24 h survival, which were compared using Fisher's exact test. The times to therapy administration were compared using a two-tailed *t*-test in Graphpad Prism 5 (GraphPad Software Inc.).

A predetermined *α* level of 0.05 was taken to indicate a statistically significant difference in all cases.

## 3. Results

**Figure 3.**
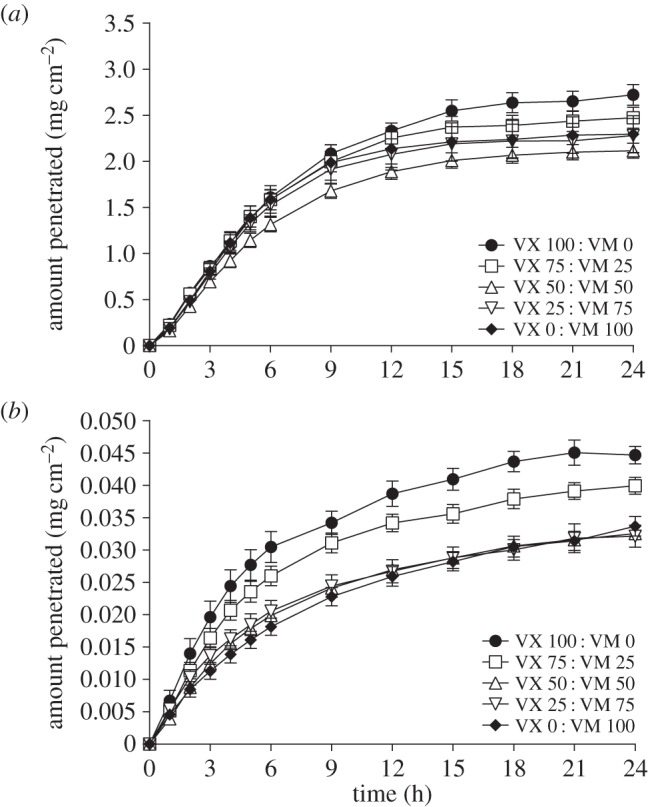
Combined penetration of ^14^C VX and ^14^C VM through guinea pig skin from matched experiments when applied neat (*a*) or diluted in IPA (*b*). There was no significant difference between the combined penetration rates of either neat or dilute agent mixtures through guinea pig skin. Points are mean±s.d.

The penetration rates of ^14^C VM or ^14^C VX from neat or dilute agent mixtures were determined in guinea pig and pig skin *in vitro* (tables 1 and 2). In guinea pig skin, there was no significant difference between the percentage of applied dose penetration rates of ^14^C VM or ^14^C VX from neat or dilute agent mixtures (table 1). At 24 h the majority of radioactivity (whether ^14^C VM or ^14^C VX) was located in the receptor fluid and guinea pig skin with only residual amounts located on the skin surface. Recoveries were similar for each mixture ratio with total recoveries in excess of 90% (neat agent studies) or 70% (dilute agent studies) of the applied dose of ^14^C. The combined penetration of ^14^C VM and ^14^C VX through guinea pig skin from matched experiments is shown in figure 3. There was no significant difference between the combined penetration rates of either neat or dilute agent mixtures through guinea pig skin.

**Table 1.** Maximum penetration rates ($J_{\max}$) expressed as g cm^−2^ h^−1^×10^−6^ and as percentage of applied dose for 10 μl of neat (*a*) and dilute (*b*) nerve agent mixture application to guinea pig skin. $J_{\max}$ determinations were made between 2 and 4 h where correlation coefficients were greater than 0.99. There was no statistical difference in the per cent of applied dose $J_{\max}$ for either VX or VM ratio penetration. The combined $J_{\max}$ value gives the penetration rate of both nerve agents present at each ratio. All values are mean±s.d. of *n*=8 diffusion cells (*n*=16 diffusion cells for combined $J_{\max}$). n.a., not applied.

guinea pig skin studies					
ratio VX:VM	$J_{\max}$ VX (g cm^−2^ h^−1^×10^−6^)	$J_{\max}$ VX (% of applied dose per hour)	$J_{\max}$ VM (g cm^−2^ h^−1^×10^−6^)	$J_{\max}$ VM (% of applied dose per hour)	combined $J_{\max}$ (g cm^−2^ h^−1^×10^−6^)
(*a*) neat agent application, total combined volume 10 μl					
100:0	260±73	6.60±1.87	n.a.	n.a.	260±73
75:25	207±84	7.02±2.83	81±21	8.24±2.09	288±75
50:50	113±32	5.72±1.62	135±32	6.88±1.62	248±51
25:75	65±26	6.65±2.69	231±50	7.82±1.69	296±54
0:100	n.a.	n.a.	312±84	7.93±2.13	312±84
(*b*) dilute agent application, total combined volume 10 μl of 22 mg ml^−1^ agent in IPA					
100:0	5.23±0.95	6.03±1.09	n.a.	n.a.	5.23±0.95
75:25	4.06±0.43	6.24±0.67	0.61±0.26	2.80±1.21	4.66±0.58
50:50	2.25±0.56	5.19±1.29	1.14±0.46	2.62±1.06	3.39±0.88
25:75	1.24±0.23	5.72±1.06	1.73±0.46	2.67±0.71	2.97±0.46
0:100	n.a.	n.a.	2.70±0.55	3.11±0.64	2.70±0.55

**Table 2.** Maximum penetration rates ($J_{\max}$) expressed as g cm^−2^ h^−1^×10^−6^ and as percentage of applied dose for 10 μl of neat (*a*) and dilute (*b*) nerve agent mixture application to pig skin. $J_{\max}$ determinations were made between 9 and 15 h where correlation coefficients were greater than 0.99. There was no statistical difference in the per cent of applied dose $J_{\max}$ for either VX or VM ratio penetration. The combined $J_{\max}$ value gives the penetration rate of both nerve agents present at each ratio. All values are mean±s.d. of *n*=8 diffusion cells (*n*=16 diffusion cells for combined $J_{\max}$). n.a., not applied.

pig skin studies					
ratio VX:VM	$J_{\max}$ VX (g cm^−2^ h^−1^×10^−6^)	$J_{\max}$ VX (% of applied dose per hour)	$J_{\max}$ VM (g cm^−2^ h^−1^×10^−6^)	$J_{\max}$ VM (% of applied dose per hour)	combined $J_{\max}$ (g cm^−2^ h^−1^×10^−6^)
(*a*) neat agent application, total combined volume 10 μl					
100 : 0	40.6±26.2	1.03±0.67	n.a.	n.a.	40.6±26.2
75 : 25	28.7±14.2	0.97±0.48	8.5±2.7	0.86±0.28	37.2±16.3
50 : 50	13.6±7.0	0.69±0.36	18.3±6.7	0.93±0.34	31.9±12.1
25 : 75	8.4±5.5	0.82±0.56	31.6±12.5	1.07±0.42	39.7±12.2
0 : 100	n.a.	n.a.	31.9±14.3	0.81±0.36	31.9±14.3
(*b*) dilute agent application, total combined volume 10 μl of 22 mg ml^−1^ agent in IPA					
100: 0	0.74±0.43	0.86±0.50	n.a.	n.a.	0.74±0.43
75 : 25	0.57±0.29	0.88±0.44	0.18±0.07	0.85±0.31	0.76±0.27
50 : 50	0.33±0.12	0.77±0.28	0.35±0.16	0.81±0.38	0.68±0.21
25 : 75	0.20±0.10	0.93±0.44	0.66±0.23	1.02±0.35	0.86±0.27
0 : 100	n.a.	n.a.	0.89±0.52	1.03±0.60	0.89±0.52

**Figure 4.**
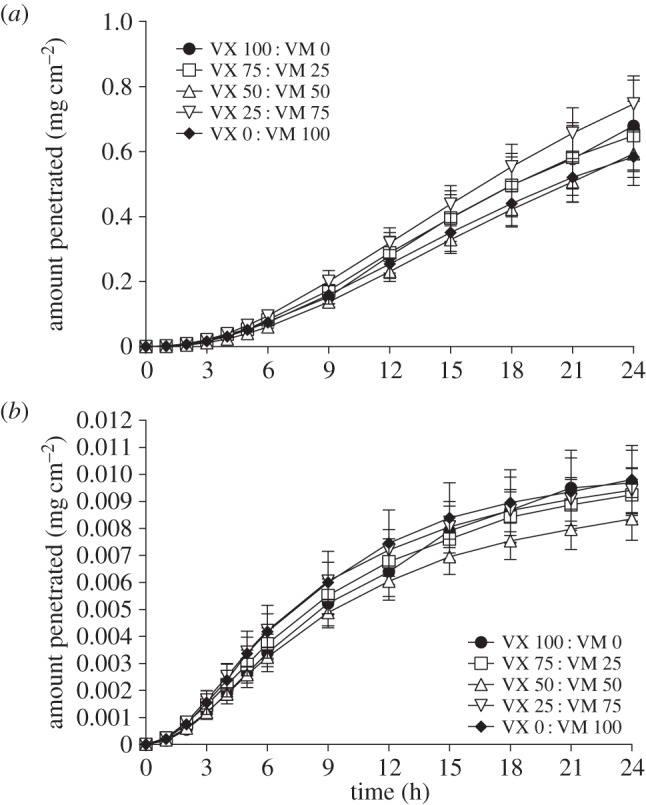
Combined penetration of ^14^C VX and ^14^C VM through pig skin from matched experiments when applied neat (*a*) or diluted in IPA (*b*). There was no significant difference between the combined penetration rates of either neat or dilute agent mixtures through pig skin. Points are mean±s.d.

In pig skin, there was no significant difference between the percentage of applied dose penetration rates of ^14^C VM or ^14^C VX from neat or dilute agent mixtures (table 2). At 24 h the majority of radioactivity for the neat studies (whether ^14^C VM or ^14^C VX) was located in the pig skin with smaller amounts located on the skin surface and in the receptor fluid. For the dilute study, similar amounts of radioactivity were located in the skin and receptor fluid. Recoveries were similar for each mixture ratio, with total recoveries in excess of 60% (neat agent studies) or 30% (dilute agent studies) of the applied dose of ^14^C. The combined penetration of ^14^C VM and ^14^C VX through pig skin from matched experiments is shown in figure 4. There was no significant difference between the combined penetration rates of either neat or dilute agent mixtures through pig skin. Guinea pig skin was more permeable to either agent than pig skin, in agreement with previous findings [26]. The penetration rate of VM was not affected by the presence of VX in mixtures of various proportions (figures 3 and 4).

**Figure 5.**
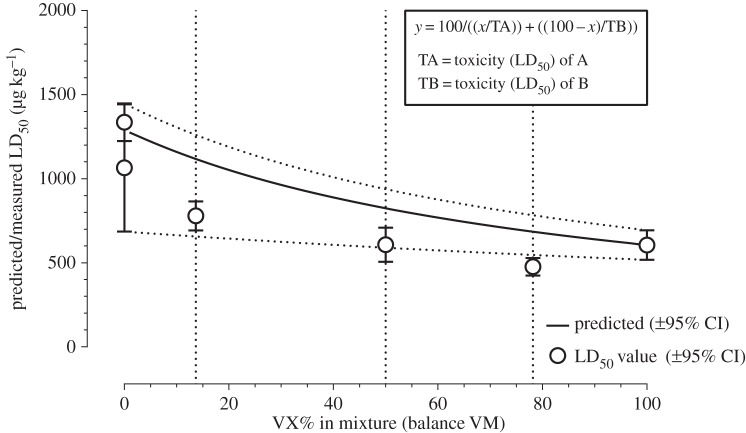
Toxicity determination (LD_50_ values) for VM alone and in three proportions with VX, applied via the percutaneous route in guinea pigs. IPA was used as the carrier solvent throughout. The error bars on the data points represent the 95% confidence interval (CI) on the fit to the data. The toxicity of VX alone was not determined in this series of experiments but was taken from a recent determination in our laboratory that used the same methodology (H Mumford & ME Price 2012, unpublished).

In guinea pigs, by the percutaneous route, the LD_50_ of VM in IPA carrier solvent was initially determined to be 1.194 mg kg^−1^ (95% CI of the probit fit to the data 0.961–1.426) and this was the estimate used to set the challenge dose for the MedCM study. The toxicity was repeated as part of the mixture series and that determination gave an estimated LD_50_ of 1.336 mg kg^−1^ (1.224–1.448, 95% CI). Re-analysis of the first data set at the conclusion of the study gave a revised estimate of 1.064 mg kg^−1^ (0.686–1.443, 95% CI) and the combined estimate from both data sets was 1.2899 mg kg^−1^ (1.199–1.381, 95% CI). This value is approximately twice that of the LD_50_ of VX (0.613 mg kg^−1^, 0.551–0.675, 95% CI). The toxicity of three mixed solutions of VM was not significantly different from the pure agents (figure 5) and was not significantly different from the theoretical predicted toxicity of the mixture. This implies that there was no interaction between the agents, either on the skin or in the body, which changed their toxicity. None of the observed signs of poisoning and post-mortem findings suggested that VM was different from other anticholinesterase nerve agents recently characterized in this model [33].

**Figure 6.**
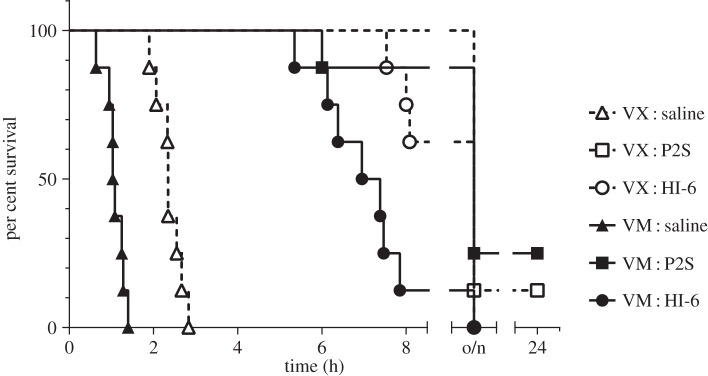
Survival in guinea pigs following percutaneous challenge with VM (2.388 mg kg^−1^) or VX (1.226 mg kg^−1^) and MedCM administered on appearance of signs of poisoning, followed by two further doses on recurrence or worsening of signs. For therapy doses see text. Animals (*n*=8 per curve) were not observed overnight, from 8 h to 22.5 h following agent application; o/n denotes animals that died overnight.

Three doses of therapy containing atropine, Avizafone and P2S or HI-6 were not sufficient to protect guinea pigs fully at 24 h from 2×LD_50_ of either VM or VX. Divided therapy, administered on the appearance of signs of poisoning and subsequently on worsening signs, prolonged the times to death compared to saline-treated controls (figure 6). At 6 h, survival was significantly higher in treated groups than in control groups for both agents and either treatment. Guinea pigs dosed with VM (2.388 mg kg^−1^) showed observable signs of systemic cholinergic poisoning, requiring administration of the first therapy dose at 54.5±2.2 min (mean±s.e.m.; *n*=24); this was significantly earlier than VX-treated animals which were dosed at 93.9±5.3 min (mean±s.e.m.; *n*=24). Note that although these doses were equitoxic, a greater mass of agent was applied in the case of VM.

## 4. Discussion

VM is a structural variant of VX with similar physicochemical properties. The synthesis of ^14^C-labelled VM, percutaneous toxicity of VM, the toxicity of mixtures of V-agents, the protection afforded by in-service medical countermeasures against a lethal percutaneous dose of VM and the skin penetration characteristics of VM alone or of mixtures of VM and VX have not been reported previously. Complementary *in vitro* and *in vivo* studies were completed to inform the advice that could be provided to those agencies undertaking the destruction of the precursor chemicals to these agents.

This study determined the penetration rate of ^14^C VM through pig and guinea pig skin *in vitro* for the first time. Guinea pig skin was more permeable than pig skin to both ^14^C VM and ^14^C VX, either alone or in combination, dilute or neat. This finding agrees with previous results for ^14^C VX penetration [26]. The penetration rate of neat ^14^C VM was not significantly different from that of neat ^14^C VX through either guinea pig or pig skin. ^14^C VM had similar penetration rates and 24 h dose distribution to that of ^14^C VX (data not shown). The presence of VX did not affect the penetration rate of ^14^C VM in mixtures of various proportions, and *vice versa*. The presence of the solvent IPA did not affect the penetration of either agent from the mixtures.

VM was approximately half as potent as VX in terms of lethality, in guinea pigs challenged via the percutaneous route. The toxicity of three mixed solutions of VM and VX was determined, and no mixture was more toxic than the most toxic component (VX). The implication from this is that there was no interaction between the agents, either on the skin or in the body, which altered their toxicity.

Although extrapolation from the guinea pig to the human situation is far from ideal, the inclusion of pig skin in the *in vitro* study increased confidence in the extrapolation. Previous work determined the penetration rates of dilute ^14^C VX (22 mg ml^−1^ in IPA) through guinea pig, pig and human skin [26]; these were 3.69±0.72, 0.73±0.35 and 1.01±0.21 μg cm^−2^ h^−1^ (mean±s.d.), respectively. The values measured during this study for the penetration rates of dilute (22 mg ml^−1^ in IPA) ^14^C VX though guinea pig and pig skin were 5.23±0.95 and 0.74±0.43 μg cm^−2^ h^−1^ (mean±s.d.), respectively. The similarity of results between these studies, and the inclusion of human skin in the previous work, gives confidence that the results of the present studies can be reliably extrapolated to human percutaneous exposures. The finding that VX and VM mixture penetration was not increased in comparison to either VX or VM penetration through either guinea pig or pig skin gives confidence that no penetration enhancement through human skin would likely be observed.

Three doses of MedCM containing atropine, Avizafone and either P2S or HI-6 were not sufficient to protect guinea pigs fully at 24 h from a 2×estimated LD_50_ of either VM or VX. Similar results have been reported by Joosen *et al.* [14]. Extrapolation of these results to humans should be treated cautiously. However, given that treatment, on appearance of poisoning and on worsening signs of poisoning, significantly prolonged the time to death, this could allow sufficient time for a casualty to receive further medical support and decontamination if the same were true for contaminated personnel [15].

## 5. Conclusion

The percutaneous penetration rate of VM has been measured for the first time *in vitro* through guinea pig and pig skin, both alone and in combination with VX. This is the first time that the penetration characteristics of VM–VX mixtures have been studied in this *in vitro* system. The percutaneous penetration rate of VM was not affected by the presence of VX and vice versa.

This study has characterized the toxicity of VM in guinea pigs, alone and in combination with VX; this is one of the few reports of toxicity determinations of mixed solutions of any organophosphorus nerve agents [34,35], and the first to our knowledge on mixtures of V-agents. A lethal dose of VM was approximately twice that of VX in our guinea pig model of percutaneous nerve agent poisoning. There was no interaction in mixed agent solutions which altered the toxicity of the agents. Percutaneous poisoning by VM or VX was responsive to treatment with standard MedCM, although complete protection was not achieved.

The penetration rate of V-agents would be slower in humans than guinea pigs (more comparable with the rate in pig skin). In the event of a confirmed exposure to either agent, decontamination and medical monitoring could be initiated as soon as practicable. It may be, however, that the onset of signs of cholinergic poisoning would be the first indication of exposure (e.g. through liquid pickup). In that case, the administration of MedCM would provide an extended ‘window of opportunity’ for decontamination and for a casualty to be evacuated and receive further medical treatment.

The results indicate that, provided the effects found *in vitro* in guinea pig and pig are reproduced in humans, there is no requirement to handle mixtures of VM and VX (or mixtures of precursors **1** with **2** and/or **3**) any differently from the pure nerve agents.

## Supplementary Material

Supplementary figures

## Data accessibility

Datasets and additional figures supporting this article have been uploaded as electronic supplementary material.
