# Supplementary material for: Toxicity and medical countermeasure studies on the organophosphorus nerve agents VM and VX
Source: Proc Math Phys Eng Sci. 2015 Apr 8;471(2176):20140891. doi: 10.1098/rspa.2014.0891 (PMC4991253; doi:10.1098/rspa.2014.0891)
Supplement: Supplementary figures [file rspa20140891supp1.docx]

Rice et al, Proc. Roy. Soc. A, 2015

Toxicity and medical countermeasure studies on the organophosphorus nerve agents VM and VX

Supplementary Figures

**Figure S1:** Penetration of **neat ^14^C VM through guinea pig skin** when applied alone or as a mixture with VX expressed as amount penetrated (mg.cm^-2^) (Top) and as % of applied dose (Bottom).

Each skin received a discrete 10µl droplet of nerve agent at a ratio of 100:0, 75:25, 50:50 or 25:75 VM to VX. All values are mean ± standard error of n=8 skins from individual animals.

**Figure S2:** Penetration of **neat ^14^C VX through guinea pig skin** when applied alone or as a mixture with VM expressed as amount penetrated (mg.cm^-2^) (Top) and as % of applied dose (Bottom).

Each skin received a discrete 10µl droplet of nerve agent at a ratio of 100:0, 75:25, 50:50 or 25:75 VX to VM. All values are mean ± standard error of n=8 skins from individual animals.

**Figure S3:** Penetration of **dilute ^14^C VM through** **guinea pig skin** when applied alone or as a mixture with VX expressed as amount penetrated (mg.cm^-2^) (Top) and as % of applied dose (Bottom).

Each skin received a discrete 10µl droplet of IPA diluted nerve agent at a ratio of 100:0, 75:25, 50:50 or 25:75 VM to VX. All values are mean ± standard error of n=8 skins from individual animals.

**Figure S4:** Penetration of **dilute ^14^C VX through guinea pig skin** when applied alone or as a mixture with VM expressed as amount penetrated (mg.cm^-2^) (Top) and as % of applied dose (Bottom).

Each skin received a discrete 10µl droplet of IPA diluted nerve agent at a ratio of 100:0, 75:25, 50:50 or 25:75 VX to VM. All values are mean ± standard error of n=8 skins from individual animals.

**Figure S5**: Dose distribution of ^14^C VM (top) and ^14^C VX (bottom) remaining on the surface of guinea pig skin (swab), within guinea pig skin (membrane) or penetrated through guinea pig skin (receptor fluid) upon study completion at 24 hours.

Each skin received a discrete 10µl droplet of nerve agent comprising a ratio 100:0, 75:25, 50:50 or 25:75 VM to VX (top) and VX to VM (bottom). All values are mean ± standard error of n=8 skins from individual animals.

**Figure S6:** Dose distribution of ^14^C VM (top) and ^14^C VX (bottom) remaining on the surface of guinea pig skin (swab), within guinea pig skin (membrane) or penetrated through guinea pig skin (receptor fluid) upon study completion at 24 hours.

Each skin received a discrete 10µl droplet of IPA diluted nerve agent comprising a ratio 100:0, 75:25, 50:50 or 25:75 VM to VX (top) and VX to VM (bottom). All values are mean ± standard error of n=8 skins from individual animals.

**Figure S7:** Penetration of **neat ^14^C VM through pig skin** when applied alone or as a mixture with VX expressed as amount penetrated (mg.cm^-2^) (Top) and as % of applied dose (Bottom).

Each skin received a discrete 10µl droplet of nerve agent at a ratio of 100:0, 75:25, 50:50 or 25:75 VM to VX. All values are mean ± standard error of n=8 skins from individual animals.

**Figure S8:** Penetration of **neat ^14^C VX through pig skin** when applied alone or as a mixture with VM expressed as amount penetrated (mg.cm^-2^) (Top) and as % of applied dose (Bottom).

Each skin received a discrete 10µl droplet of nerve agent at a ratio of 100:0, 75:25, 50:50 or 25:75 VX to VM. All values are mean ± standard error of n=8 skins from individual animals.

**Figure S9:** Penetration of **dilute ^14^C VM through pig skin** when applied alone or as a mixture with VX expressed as amount penetrated (mg.cm^-2^) (Top) and as % of applied dose (Bottom).

Each skin received a discrete 10µl droplet of IPA diluted nerve agent at a ratio of 100:0, 75:25, 50:50 or 25:75 VM to VX. All values are mean ± standard error of n=8 skins from individual animals.

**Figure S10:** Penetration of **dilute ^14^C VX through pig skin** when applied alone or as a mixture with VM expressed as amount penetrated (mg.cm^-2^) (Top) and as % of applied dose (Bottom).

Each skin received a discrete 10µl droplet of IPA diluted nerve agent at a ratio of 100:0, 75:25, 50:50 or 25:75 VX to VM. All values are mean ± standard error of n=8 skins from individual animals.

**Figure S11:** Dose distribution of ^14^C VM (top) and ^14^C VX (bottom) remaining on the surface of pig skin (swab), within pig skin (membrane) or penetrated through pig skin (receptor fluid) upon study completion at 24 hours.

Each skin received a discrete 10µl droplet of nerve agent comprising a ratio 100:0, 75:25, 50:50 or 25:75 VM to VX (top) and VX to VM (bottom). All values are mean ± standard error of n=8 skins from individual animals.

**Figure S12:** Dose distribution of ^14^C VM (top) and ^14^C VX (bottom) remaining on the surface of pig skin (swab), within pig skin (membrane) or penetrated through pig skin (receptor fluid) upon study completion at 24 hours.

Each skin received a discrete 10µl droplet of IPA diluted nerve agent comprising a ratio 100:0, 75:25, 50:50 or 25:75 VM to VX (top) and VX to VM (bottom). All values are mean ± standard error of n=8 skins from individual animals.

**Table S1: LD_50_ Combined Reanalysis Summary**

VX 100% determination from previous work in our laboratory (Price & Mumford, 2013, unpublished)

|  |  |  |  | **LD_50_** | | | | | | | |
| --- | --- | --- | --- | --- | --- | --- | --- | --- | --- | --- | --- |
|  |  | **Component Proportions** | | **Log Scale** | | | | **Original Scale** | | | |
| **Fitting Method** | **Mixture** | **VM** | **VX** | **Estimate** | **Std. Error** | **Lower Bound** | **Upper Bound** | **Estimate** | **Std. Error** | **Lower Bound** | **Upper Bound** |
| BRGLM | 0/100 | 0 | 100 | 2.78 | 0.0 | 2.72 | 2.85 | 605.6 | 45.0 | 517.4 | 693.8 |
| BRGLM | 22/78 | 22 | 78 | 2.68 | 0.0 | 2.63 | 2.72 | 476.2 | 26.3 | 424.7 | 527.8 |
| BRGLM | 50/50 | 50 | 50 | 2.78 | 0.0 | 2.71 | 2.86 | 607.6 | 51.6 | 506.5 | 708.7 |
| BRGLM | 86.3/13.7 | 86.3 | 13.7 | 2.89 | 0.0 | 2.84 | 2.94 | 779.1 | 44.0 | 692.8 | 865.4 |
| BRGLM | 100/0 a | 100 | 0 | 3.03 | 0.1 | 2.87 | 3.18 | 1064.4 | 193.3 | 685.6 | 1443.2 |
| BRGLM | 100/0 b | 100 | 0 | 3.13 | 0.0 | 3.09 | 3.16 | 1336.0 | 57.0 | 1224.3 | 1447.8 |
| BRGLM | 100/0 combined | 100 | 0 | 3.11 | 0.0 | 3.08 | 3.14 | 1289.9 | 46.6 | 1198.6 | 1381.1 |
